# Correlation of *Adiponectin* mRNA Abundance and Its Receptors with Quantitative Parameters of Sperm Motility in Rams 

**DOI:** 10.22074/ijfs.2016.4778

**Published:** 2016-04-05

**Authors:** Ali Kadivar, Heidar Heidari Khoei, Hossein Hassanpour, Arefe Golestanfar, Hamid Ghanaei

**Affiliations:** 1 Department of Clinical Science, Faculty of Veterinary Medicine, Shahrekord University, Shahrekord, Iran; 2 Research Institute of Biotechnology, Shahrekord University, Shahrekord, Iran; 3 Research Institute of Animal Embryo Technology, Shahrekord University, Shahrekord, Iran; 4 Faculty of Medicine, Shahid Beheshti University of Medical Sciences, Tehran, Iran

**Keywords:** *Adiponectin*, *AdipoR1*, *AdipoR2*, Sperm Motility

## Abstract

**Background:**

*Adiponectin* and its receptors (AdipoR1 and AdipoR2), known as adiponectin system, have some proven roles in the fat and glucose metabolisms. Several studies have shown that adiponectin can be considered as a candidate in linking metabolism to testicular function. In this regard, we evaluated the correlation between sperm mRNA abundance of adiponectin and its receptors, with sperm motility indices in the present study.

**Materials and Methods:**

In this completely randomized design study, semen samples from 6 adult rams were fractionated on a two layer discontinuous percoll gradient into high and low motile sperm cells, then quantitative parameters of sperm motility were determined by computer-assisted sperm analyzer (CASA). The mRNA abundance levels of *Adiponectin*, *AdipoR1* and *AdipoR2* were measured quantitatively using real-time reverse transcriptase polymerase chain reaction (qRT-PCR) in the high and low motile groups.

**Results:**

Firstly, we showed that adiponectin and its receptors (*AdipoR1* and *AdipoR2*) were transcriptionally expressed in the ram sperm cells. Using Pfaff based method qRT- PCR, these levels of transcription were significantly higher in the high motile rather than low motile samples. This increase was 3.5, 3.6 and 2.5 fold change rate for *Adiponectin*, *AdipoR1* and *AdipoR2*, respectively. Some of sperm motility indices [curvilinear velocity (VCL), straight-line velocity (VSL), average path velocity (VAP), linearity (LIN), wobble (WOB) and straightness (STR)] were also significantly correlated with *Adiponectin* and *AdipoR1* relative expression. The correlation of *AdipoR2* was also significant with the mentioned parameters, although this correlation was not comparable with adiponectin and AdipoR1.

**Conclusion:**

This study revealed the novel association of adiponectin system with sperm motility. The results of our study suggested that adiponectin is one of the possible factors which can be evaluated and studied in male infertility disorders.

## Introduction

*Adiponectin* is a member of the adipose-secreted proteins, called adipocytokines. *Adiponectin* was initially described as a 30 kDa adipocyte complementrelated protein (1). It is a 244-amino acids protein and the most abundant adipose-derived hormone secreted by adipocytes in white adipose tissue with relevant roles in lipid metabolism and glucose homeostasis (2). *Adiponectin* also plays role on stimulation of fatty acid oxidation in the liver and skeletal muscle, suppression of hepatic gluconeogenesis, stimulation of glucose uptake in skeletal muscle and increasing insulin secretion (3). Following production, the actions of adiponectin are supported by two distinct but structurally related adiponectin receptors (AdipoR), AdipoR1 and AdipoR2 (4). The metabolic importance of these receptors is now firmly established. So that, *AdipoR1*(/) and *AdipoR2*(/) mouse models exhibited various disorders due to aberration in the fat and glucose metabolisms (5). 

In addition to the well-known metabolic effects, it has been shown that adiponectin could affect the reproductive system, in part, through central actions on the hypothalamus-pituitary axis (6). Hypothalamic neurons secrete gonadotropin-releasing hormone (GnRH) with a pulsatile pattern, stimulating the release of pituitary gonadotropins, follicle-stimulating hormone (FSH) and luteinizing hormone (LH). These gonadotropins regulate testicular steroidogenesis and spermatogenesis (7). *AdipoR1* and *AdipoR2* are generally expressed in human hypothalamus and pituitary (8), so adiponectin can presumably be involved in the modulation of the endocrine reproductive axis in humans. *Adiponectin* and its receptors are also expressed by different cell types of the male gonad, suggesting a possible regulation of testicular function by adiponectin, through endocrine and/or paracrine actions. In chicken, presence of the adiponectin system (adiponectin, *AdipoR1* and *AdipoR2*) was demonstrated in peritubular and seminiferous tubule cells (9). In line with this, testicular expression of adiponectin receptors was found to be higher at mRNA or protein levels in adult compared to prepubertal chickens and rats (9,10), suggesting that sexual maturation is likely associated with an increased sensitivity to the changes in plasma adiponectin levels. A significant, positive relationship was also reported between plasma adiponectin and high-density lipoprotein cholesterol in men (11) which may contribute to testosterone production. In this regard, Laughlin et al. (12) showed that, in the men and women, serum adiponectin is positively related to testosterone and high-density lipoprotein. 

In general, the previous studies demonstrated a close relationship between adiponectin system and reproductive function. So, we hypothesized that the sperm *Adiponectin*, *AdipoR1* and *AdipoR2* mRNA abundances might correlate with sperm motility indices. 

## Materials and Methods

### Semen samples and spermatozoa preparations

In this completely randomized design, testicles of 6 adult rams were collected from an official abattoir and transferred to the laboratory at room temperature (20-25°C). All procedures to sacrifice the animals were carried out at abattoir in accordance with Iranian government rules. Semen collection was carried out within the first 2 hours after the slaughter of the ram. Epididymis-testicle complexes were dissected into two parts: testicle, epididymis. Sperm was obtained by slicing the tissue of the cauda epididymis with a scalpel; the fluid was collected by sampler and the volume was estimated. To prohibit contamination, epididymis samples were carefully dissected free of blood clots and extraneous tissues. Care was taken to no cut blood vessels. 

Semen samples were washed with Hepes-buffered tissue culture medium (Hepes TCM, Gibco, Life technologies, USA)+10% bovine serum albumin (BSA, Gibco, Life technologies, USA) and sperm suspensions were centrifuged at 500 g for 2 minutes. The supernatant was then discarded. This procedure repeated two times. The sperm of 6 rams was subsequently separated into low and high motility categories, as described below. 

### Sperm separation procedures

Sperm suspension was layered on a two-layer discontinuous Percoll gradient, consisting of 1 ml of 45% (v/v) and 2 ml of 90% (v/v) Percoll (Uppsala, Sweden) in a 15 ml conical plastic tube (Falcon No. 2095, Fisher Scientific, Pittsburg, USA). The tube was centrifuged at 700 g for 20 minutes. After centrifugation, the separated fractions in the tube were carefully transferred into a new set of tubes, and the volume of each fraction was determined. 

### Spermatozoa evaluation

The assessment of motility parameters was carried out, using computer-assisted sperm analysis (CASA, HooshmandFanavar, Iran). Samples were diluted (10- 20×10^6^ cells/ml) in the same Hepes TCM medium with 320 mOsm/kg, and kept warm in the 37°C incubator during examination. Subsequently, 5 μl sample drop was placed into a Makler counting cell chamber (20 μm depth) and evaluated. Evaluation was carried out on both groups of the separated sperm by Percoll gradient.

The CASA settings were selected as follow: 6 visionfields per sample, 20 frames per second with the time analysis of less than 15 seconds per frame, 0-180 μm/ second analysis power for sperm velocity, and magnifying power of ×4 for microscope objective lens. 

In CASA analysis results, sperm motility was divided into classes A, B, C and D as rapid motility, slow motility, non-progressive motility and immotility, respectively. Besides, the followed sperm motion parameter indices were studied: curvilinear velocity (VCL), the time-average of velocity along the actual trajectory for a spermatozoon in micrometers per second, straight line velocity (VSL) representing the average velocity of sperm from the first to last position of a sperm head in a track by micrometers per second. A straight-line path from the first to last position of a sperm head was plotted, and velocity along this trajectory was termed VSL (micrometers per second). The average path of sperm cell motion was also computed, and averagetime of velocity along the average path was calculated and named as average path velocity (VAP, micrometers per second). For each centroid location of sperm, there was a deviation from the average path, called as the amplitude of lateral head displacement (ALH, micrometers). Beat cross frequency (BCF) was the frequency of sperm cell’s head cross, through the sperm cell’s average pathway in Hertz. Similarly, there were points where the curvilinear path intersects the average path, and the number of such intersections was termed as BCF (number per second). The linearity (LIN) represents the linearity of a curvilinear path in percentage. The wobble (WOB) was the measure of the actual path oscillation with regard to the average path, and the mean angular displacement (MAD) was the average time for the instantaneous turning angle absolute values of the sperm head, along with the curvilinear trajectory in degree (13). 

### RNA extraction and cDNA synthesis of sperm cells

Total RNA isolation was carried out on sperm cells, according to the acid guanidiniumthiocyanatephenol-chloroform single-step extraction protocol, as described earlier (14). Treatment of total RNA with RNase-free DNase (Sinaclon Bioscience, Iran) was performed to avoid amplification of contaminating genomic DNA. The quality and integrity of the purified RNA was controlled by measurement of the A260/A280 nm ratio as well as agarose gel electrophoresis. Only RNA samples showing integrity by electrophoresis and exhibiting an A260/A280 ratio of >1.9 were used for synthesis of cDNA. 

Total RNA was reverse transcribed into cDNA using moloney murine leukemia virus reverse transcriptase (M-MLV RT, Sinaclon Bioscience, Iran). The reverse transcribed mixture was incubated at 75ºC for 15 minutes to denature the RNA, and then stored at -20ºC. 

### Real-time quantitative reverse transcriptasepolymerase chain reaction analysis

The levels of all three transcripts (*Adiponectin*, *AdipoR1* and *AdipoR2*) were determined by real-time reverse transcriptase polymerase chain reaction. Glyceraldehyde-3-phosphate dehydrogenase (*GAPDH*) was selected as a housekeeping gene to normalize the difference of input load of cDNA between the samples. Specific primers for cDNA of *Adiponectin*, *AdipoR1*, *AdipoR2* and *GAPDH* were designed using primer BLAST (http://www.ncbi.nlm.nih.gov/primerblast). Nucleotide sequences of the selected primer pairs and the length of amplified product are presented in the Table 1. 

**Table 1:** Characteristics of the used primers


Gene	NIH GenBank accession no.	Product length (bp)	Primer sequence 5´-3´

*GAPDH*	NM_001190390.1	116	F: GTTCCACGGCACAGTCAAGG
			R: ACTCAGCACCAGCATCACCC
*Adiponectin*	KJ159213.1	132	F: CGGCACCACTGGCAAATTCC
			R: TGGTCGTGGGTGAAGAGCAG
*AdipoR1*	KJ159212.1	131	F: CAGGGGTGCAGGAGGAACTT
			R: GTGGGCTGAAGCTTGGTTGG
*AdipoR2*	KF921623.1	155	F: GCATCGCAGCCATCATCGTC
			R: GATGGTGGCAGCCTTCAGGA


Real-time quantitative reverse transcriptasePCR (qRT-PCR) analysis was performed on Rotor-Gene Q 6000 System (Qiagen, USA) using SYBR premix EX Tag ІІ (Takara, China). One microliter of cDNA was added to the master-mix (0.5 µM of each specific primer, and 10 µl of SYBR premix EX Tag ІІReady Mix) in a total volume of 20 µl. Aliquot of each reaction mixture was analyzed by electrophoresis in 1.5% agarose gel and stained with 0.5 μg/ ml ethidium bromide. The relative quantification of three gene transcripts was determined in low and high motile sperm groups. Reaction condition was performed as 95°C for 5 minutes, 45 cycles of 95°C for 40 seconds, 63°C for 30 seconds and 72°C for 30 seconds. The PCR amplification was performed in triplicate for each sample. 

Gene expression data were normalized to *GAPDH* (as internal reference gene). Data were analyzed using LinRegPCR software version 2012.0 (Amsterdam, Netherland), to give the threshold cycle (Ct) number . Mean efficiency values (E) for each gene were also determined from the amplification profiles of individual samples with this software (15). 

The mRNA level of each target gene relative to GAPDH was estimated for each sample in two experimental groups by following formula: *E GAPDH* ^(Ct high motile)^/*E Adiponectin*^(Ct high motile)^. Then, the comparison was statistically done between groups. To determine fold change for each gene, the relative gene expression of high motile group relative to low motile group were calculated as following (16,17). 

$$\mathrm{Ratio}=\frac{E_{\mathrm{GAPDH}}^{\mathrm{(Ct high motile)}}}{E_{\mathrm{Adiponectin}}^{\mathrm{(Ct high motile)}}}÷\frac{E_{\mathrm{GAPDH}}^{\mathrm{(Ct low motile)}}}{E_{\mathrm{Adiponectin}}^{\mathrm{(Ct low motile)}}}$$

To ensure product homogeneity, the melting curve analysis was performed after the real-time PCR procedure. The fluorescence signals were recorded continuously during temperature ramp (65-95°C). 

## Statistical analysis

Differences between experimental group means were analyzed through paired t test with SPSS, version 16.0 (SPSS Inc., USA). The Pearson correlation procedure was used to evaluate correlation between the level of mRNA abundance and all quantitative sperm motion parameters for the indicated genes. All results are shown as mean ± SEM and differences were considered significant at P< 0.05. 

## Results

**Fig.1:**
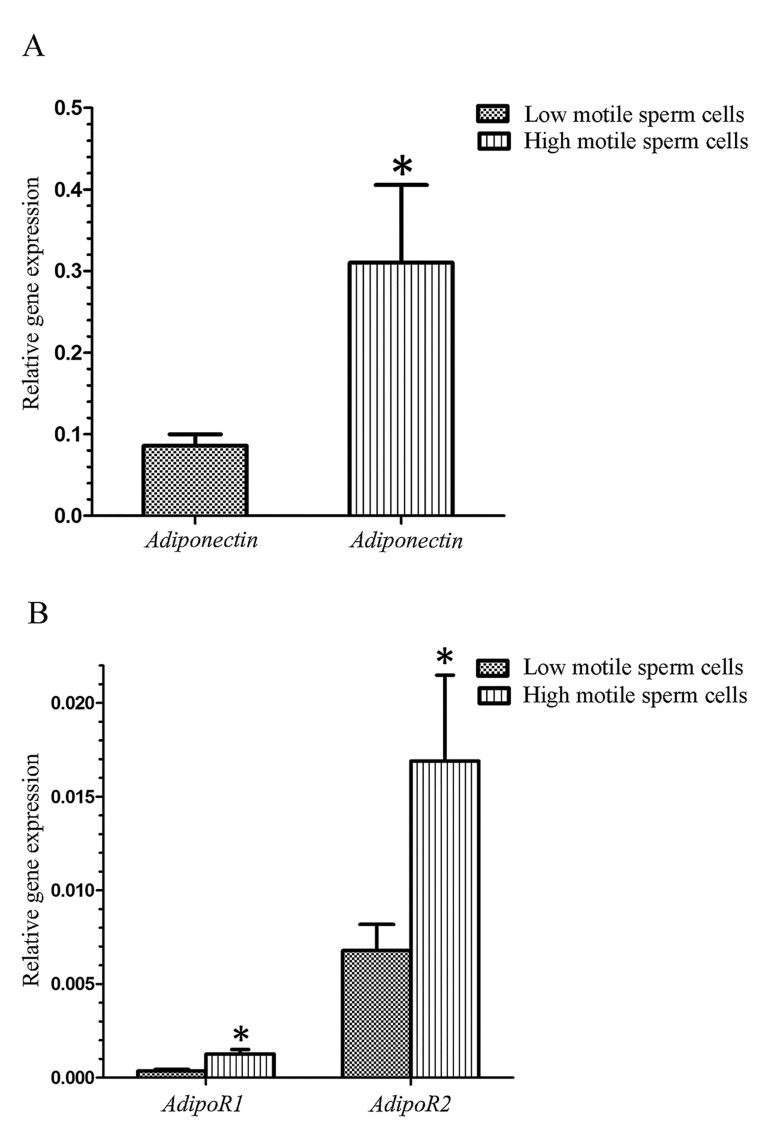
Relative abundance of A. *Adiponectin*, B. *AdipoR1* and *AdipoR2* mRNA in low and high motile sperm groups. *; Significant difference between two groups.

The results of CASA evaluation for sperm motility and sperm motility pattern are given in the Tables 2 and 3. After separation on Percoll gradient, the remaining sperm phase, in 45% Percoll, had significantly lower motile sperm cells (Table 2). The high motile sperm groups were also significantly better in other sperm motility parameters, such as VCL, VSL, VAP, LIN, WOB and STR (Table 3). This result showed that separation procedure was performed well. After separation, we analyzed the mRNA abundance of three genes between high and low motile sperm groups. As presented in the Figure 1, the mean level of *Adiponectin*, *AdipoR1* and *AdipoR2* gene abundances was significantly higher in high motile group than low motile. In the next step and for more evaluation, the correlation analysis was performed between the level of mRNA abundance and all sperm motion parameters for all three genes in motile and immotile sperm groups. In this analysis, all samples from high and low motile groups were considered together and the general correlation between motility indices and the level of mRNA abundance was calculated. The results of this analysis showed that mRNA abbundance for *Adiponectin* gene had a significant positive correlation with the class A of sperm motility, percent of progressive motile sperms, percent of motile sperms, VCL, VSL, VAP, LIN and WOB (Table 4). The amount of mRNA for *AdipoR1* gene also showed a significant positive correlation with class A of sperm motility, percent of progressive motile sperms, LIN, WOB and STR (Table 4). For *AdipoR2* gene, significant correlation was only observed with WOB. 

**Table 2:** Concentration, motility and progression of Percoll separated sperm samples (evaluated by CASA). Results are given as mean ± SE


Groups	Sperm density (Mill/ml)	Motile sperm (%)	Progression (%)			
			Fast progressive (class A)	Slow progressive (class B)	Non progressive (class C)	Non motile (class D)

High motile (n=6)	12.07 ± 2.56^ns^	76.40 ± 2.27**	58.53 ± 3.52****	12.01 ± 4.66^ns^	5.85 ± 0.51^ns^	23.60 ± 2.2**
Low motile (n=6)	13.46 ± 1.73	58.49 ± 4.47	29.36 ± 2.41	16.34 ± 6.67	9.01 ± 3.85	44.00 ± 3.84


CASA; Computer-assisted sperm analyzer, ^ns^; Not significant, **; P<0.01 and ****; P<0.0001.

**Table 3:** Sperm motility pattern parameters of percoll separated sperm samples (evaluated by CASA). Results are given as mean ± SE


Groups	Sperm motility pattern								
	VCL(μm/s)	VSL(μm/s)	VAP(μm/s)	MAD(%)	ALH(%)	BCF (%)	LIN (%)	WOB (%)	STR (%)

High motile (n=6)	80.93 ± 9.66*	57.13 ± 8.47**	65.58 ± 8.66*	20.20 ± 3.28 ns	3.03 ± 0.18 ns	2.49 ± 0.77 ns	56.53 ± 2.56**	71.24 ± 1.59***	71.52 ± 1.95**
Low motile (n=6)	49.75 ± 7.78	23.65 ± 3.06	32.39 ± 5.66	13.86 ± 3.18	2.67 ± 0.26	1.73 ± 0.75	36.31 ± 3.48	54.64 ± 2.66	57.19 ± 2.97


CASA; Computer-assisted sperm analyzer, VCL; Curvilinear velocity, VSL; Straight-line velocity, VAP; Average path velocity, MAD; Mean angular displacement, ALH; Amplitude of lateral head displacement, BCF; Beat cross frequency, LIN; Linearity, WOB; Wobble, STR; Straightness, ^ns^; Not significant, *; P<0.05, **; P<0.01 and ***; P<0.001.

**Table 4:** Correlations between the amount of relative gene abundance for Adiponectin, AdipoR1 and AdipoR2 with quantitative sperm motion parameters


Groups	Sperm motility pattern											
	MS(%)	Class A(%)	SPM(%)	VCL(μm/s)	VSL(μm/s)	VAP(μm/s)	MAD(%)	ALH(%)	BCF (%)	LIN (%)	WOB (%)	STR (%)

Adiponectin relative abundance	0.51*	0.66*	0.56*	0.54*	0.60**	0.61**	-0.10^ns^	0.03^ns^	0.18^ns^	0.53*	0.64**	0.06 ^ns^
AdipoR1 relative abundance	0.19^ns^	0.44*	0.44*	0.47^ns^	0.19^ns^	0.23^ns^	0.67^ns^	-0.014^ns^	0.56^ns^	0.46*	0.55**	0.47*
AdipoR2 relative abundance	0.21^ns^	0.36^ns^	0.26^ns^	0.10^ns^	0.15^ns^	0.16^ns^	-0.03^ns^	0.11^ns^	0.28^ns^	0.32^ns^	0.45*	0.26^ns^


MS; Motile sperm; Class A; Sperm with fast progressive motility, SPM; Sperm with progressive motility (class A+class B), VCL; Curvilinear velocity, VSL; Straight-line velocity, VAP; Average path velocity, MAD; Mean angular displacement, ALH; Amplitude of lateral head displacement, BCF; Beat cross frequency, LIN; Linearity, WOB; Wobble, STR; Straightness, ^ns^; Not significant, *; P<0.05 and **; P<0.01.

## Discussion

In the present study, associations between sperm mRNA abundance of *Adiponectin* and its receptors, *AdipoR1* and *AdipoR2*, with quantitative parameters of sperm motility were evaluated in rams. *Adiponectin* is a plasma protein with about 0.01% of total serum proteins concentration (18). The primary amino acid sequences of *Adiponectin* are highly conserved across the species (19). For example, bovine adiponectin shows 92% homology with human *Adiponectin* and 82% homology with murine *Adiponectin* (20). *Adiponectin* is synthesized as a single monomer which undergoes multimerization to provide three multimer forms with different molecular weights (MWs): i. Low molecular weight (LMW) *Adiponectin* composed of three monomers that are combined to form a trimmer, ii. Middle molecular weight (MMW) *Adiponectin*, as a hexamer formed by two trimmers, and iii. High molecular weight (HMW) multimer of *Adiponectin*, comprised of 12-18 monomers. 

Ejaculated sperm retains a complex and specific population of RNAs. It was recently proposed that these RNA molecules may have important roles in the sperm development, chromatin repackaging and even zygote development (21). Studies on sperm RNA are available for humans (22), stallion (23), cattle (24) and boars (25). Analysis of the mRNA profiles in the normal and abnormal sperms is a growing field, which can become a diagnostic and prognostic tool to evaluate male fertility and can eventually lead to identify specific genetic pathways which are necessary for production of the fertile sperm. For example, studies have been conducted to compare the genetic profiles of sperm samples from normal fertile men and teratozoospermic patients (26,27). 

In the present study, the mRNA abundances of all three components of adiponectin system were significantly higher in the high motile sperm groups. The mRNA abundances also positively correlated with some of the most important parameters of sperm motility pattern, especially for *Adiponectin* and *AdipoR1*. *Adiponectin* and the relevant receptors play major roles in sperm morphology and function, contributing to increase fertility. A recent study by Kasimanickam et al. (28) showed that *Adiponectin*, *AdipoR1*, and *AdipoR2* were immunolocalized in the acrosomal, postacrosomal, equatorial, and tail regions of bull sperm. In this study, serum *Adiponectin* concentration and sperm mRNA expressions for *Adiponectin* and its receptors showed a significant positive correlation with sire conception rate. In ram, transcripts for adiponectin system components, have been detected in the testis, all parts of epididymis, vesicular and bulbourethral glands (29). Expression of *Adiponectin* receptors was also reported in porcine epididymis (30). 

Our results showed a novel evidence for the presence of *Adiponectin* and its two receptors in at least sperm cells from cauda-epididymides. In this context, Rahmanifar and Tabandeh (29) also determined that *Adiponectin*, *AdipoR1* and *AdipoR2* were expressed in all parts of epididymides (caput, corpus, and cauda), while *AdipoR2* mRNA expression was higher than *AdipoR1*. These results, in addition to the finding reported by Kasimanickam et al. (28) indicating that gene expression of *Adiponectin* and its receptors during preand post-capacitation in spermatozoa, provide evidences of possible production of fertile sperm by local actions of *Adiponectin* at the testis level. 

In this regard, it should be noted that a spermspecific ATP-binding cassette (ABC) transporter regulates intracellular lipid metabolism in rodents (31). Kitajima et al. (32) showed that *Adiponectin* and its receptors (*AdipoR1* and *AdipoR2*) increased cholesterol efflux and reconstituted high-density lipoprotein-induced efflux, at least partially through an ABCA1 pathway. In that study, *AdipoR1*and *AdipoR2*-transfected cells showed greater cholesterol efflux when treated with *Adiponectin*. In contrast, down-regulation of adiponectin receptors decreased reconstituted high-density lipoprotein-induced cholesterol efflux. *Adiponectin* related signaling pathways in the sperm cell are not well studied until now. But *Adiponectin* and its receptors might participate in cholesterol efflux via a sperm-specific ABC transporter and thereby affect sperm hyperactivation and capacitation (33). 

The positive correlation of obesity with male infertility could be the evidence of clinical importance of *Adiponectin* in the fertility. In rodents, the obesity leads to sub-fertility caused by reduced sperm motility (34). Obese men have also reduced sperm concentration and total sperm count (35). In an interesting study performed by Thomas et al. (36), normal-weight men showed higher concentrations of *Adiponectin* in the seminal plasma and blood serum. In addition, *Adiponectin* concentration in seminal plasma positively correlated with sperm concentration and normal morphology of spermatozoa. Hammoud et al. (37) also found that asthenozoospermia and oligozoospermia were increased due to high body-mass index and worsened from overweight to obese men. One possible reason can be the correlation between plasma *Adiponectin* and testosterone concentrations. In this regard, Ribot et al. (38) confirmed that the diet-induced obesity in rats leads to decrease in the effective production of *Adiponectin*. Studies showed that *Adiponectin* played roles in gonadal steroidogenesis. As a paralog of *Adiponectin*, CTRP3 (a member of the C1q/TNF-related protein superfamily) was expressed at high level in the adipose tissue. In adult mouse testis, CTRP3 was expressed in Leydig cells and contributes to increase testosterone production by up-regulating Cyp11A1 and Star protein expressions (39). Interestingly, *Adiponectin* has been shown to regulate the expression of steroidogenic genes (*Star, Cyp11Aa1* and *Cyp19A1*) in human, rat, chicken and swine ovary (40,41), suggesting that *Adiponectin* might affect steroidogenesis in Leydig cells through regulation of steroidogenic gene expressions as well. This finding was also confirmed by Erdemir et al. (42). In this study, the male fat rats, had significantly lower levels of testosterone compared to the controls. In terms of pathologic evaluation, Johnsen Score (a 1-10 degree score for microscopic evaluation of spermatogenesis quality in testicular tissue) was significantly lower in the fat male rats. 

## Conclusion

The findings indicated that the products of *Adiponectin* gene may be involved in the physiology of sperm cell movement. Although the exact role of *Adiponectin* in the male reproductive system remains hypothetical, demonstrated expression of this gene in epididymal spermatozoa in this study and all parts of epididymidis suggests a possible role of *Adiponectin* in maturational spermatozoa changes, as they transit the duct. 

Moreover, considering the demonstrated correlation between obesity and fertility impairment, the results of such studies will help to find the molecular mechanisms involved in the pathogenesis of this disorder. 
